# High bone fracture risk in a large modern cohort of liver transplant recipients

**DOI:** 10.1007/s11739-024-03767-5

**Published:** 2024-09-27

**Authors:** Guido Zavatta, Giovanni Vitale, Maria Cristina Morelli, Paolo Pianta, Laura Turco, Federica Mirici Cappa, Matteo Ravaioli, Matteo Cescon, Fabio Piscaglia, Paola Altieri, Uberto Pagotto, BOLTBO study group, Francesca Caputo, Luca Vizioli, Sonia Berardi, Vittoria Vero, Anna Baldan, Roberto Di Donato, Letizia Anna Marcantoni, Mariachiara Corrado

**Affiliations:** 1 Division of Endocrinology and Diabetes Prevention and Care, IRCCS Azienda Ospedaliero-Universitaria Di Bologna, Bologna, Italy; 2 Department of Medical and Surgical Sciences (DIMEC), Alma Mater Studiorum University of Bologna, Bologna, Italy; 3 Internal Medicine Unit for the Treatment of Severe Organ Failure, IRCCS Azienda Ospedaliero-Universitaria Di Bologna, Bologna, Italy; 4 Hepatobiliary and Transplant Surgery Unit, IRCCS Azienda Ospedaliero-Universitaria Di Bologna, Bologna, Italy; 5 Division of Internal Medicine, Hepatobiliary and Immunoallergic Diseases, IRCCS Azienda Ospedaliero-Universitaria Di Bologna, Bologna, Italy; 6 IRCCS Azienda Ospedaliero-Universitaria Di Bologna, Bologna, Italy

**Keywords:** Liver transplantation, Cirrhosis, Liver failure, Vertebral fractures, Osteoporosis

## Abstract

Liver transplantation (LT) has historically been associated with a high prevalence of osteoporosis, but most of the available data date back to late 1990s–early 2000s with limited sample size. Our aim was to assess the prevalence of bone fragility fractures and contributing factors in a large modern cohort of liver transplant recipients. Retrospective study of 429 consecutive patients receiving liver transplantation from 1/1/2010 to 31/12/2015. Final cohort included 366 patients. Electronic radiological images (lateral views of spine X-rays or Scout CT abdominal scans) performed within 6 months from LT, were blinded reviewed to screen for morphometric vertebral fractures. Symptomatic clinical fragility fractures were recorded from the medical records. Patients with fragility fractures in the cohort were 155/366 (42.3%), with no significant differences between sexes. Most sustained vertebral fractures (145/155, 93.5%), mild or moderate wedges, with severe fractures more frequently observed in women. Multiple vertebral fractures were common (41.3%). Fracture rates were similar across different etiologies of cirrhosis and independent of diabetes or glucocorticoids exposure. Kidney function was significantly worse in women with fractures. Independently of age, sex, alcohol use, eGFR, and etiology of liver disease, low BMI was significantly associated with an increased risk for fractures (adjusted OR 1.058, 95%CI 1.001–1.118, *P* = 0.046). Our study shows a considerable fracture burden in a large and modern cohort of liver transplant recipients. Given the very high prevalence of bone fractures, a metabolic bone disease screening should be implemented in patients awaiting liver transplantation.

**Supplementary Information:**

The online version contains supplementary material available at 10.1007/s11739-024-03767-5.

## Introduction

Osteopenia, osteoporosis, and fragility fractures are frequent complications of patients with chronic liver disease and cirrhosis. The prevalence of metabolic bone disease in patients with cirrhosis is estimated at 12–55%, higher than the general population of same age [1, 2], and up to 40% of patients with chronic liver disease may experience fragility fractures [3, 4]. This prevalence is even higher in patients with hemochromatosis or cholestatic liver diseases in whom [5] the bone histomorphometry can reveal a specific cholestatic osteopenia, characterized by elevated bone resorption and decreased bone formation [6].

Over the last years, the average age of liver transplantation (LT) recipients has progressively increased with a shift from viral to metabolic etiology of the underlying liver disease, with a correlated increase of LT carriers’ comorbidities, chronic medications, and higher waitlist post-transplant mortality [7]. Although post-transplant bone and mineral disorders are associated with increased morbidity and mortality, only few studies, with limited sample size and dating back to the late 1990s–early 2000s, have shown that the prevalence of osteoporosis could be related to the severity of liver disease in the transplant setting [8] and that cholestatic etiology, female sex and lower body weight are important predictors of osteoporosis [9].

The most common fracture site in patients with chronic liver disease has been reported to be vertebrae with fractures of the femoral neck relatively uncommon [10]. Since up to one-third of vertebral fractures can be asymptomatic, it has been suggested that spine X-rays is an essential tool in the clinical assessment of patients with secondary osteoporosis [10].

In patients with chronic liver disease, the balance in bone remodeling activities between osteoclasts and osteoblasts is profoundly altered by the liver disease [11]. Guidelines provided by most osteoporosis societies describe the causes of osteoporotic fractures in patients with chronic liver disease as a result of nutritional deficiencies due to the underlying organ disease [5, 12]. However, several studies have suggested that osteoporosis in cirrhotic patients is a multifactorial disease in which different mechanisms act to deteriorate bone mass, thus determining bone fragility [3]. Several etiologies may determine chronic liver disease, with different pathogenetic mechanisms [5]. For example, while hemochromatosis and cholestatic diseases are respectively characterized by significant increase in iron and bilirubin, which cause osteoblast inhibition [13], by contrast, viral hepatitis is associated with an activation of the immune response and cytokine release which in turn stimulate bone resorption [10, 14, 15]. Overall, two main pathophysiological mechanisms underlying osteoporosis in patients with chronic liver disease have been thus far recognized, similar to primary osteoporosis: decreased bone formation or increased bone resorption, or both.

Considering the significantly outdated and small sample-sized literature on this topic, as well as the lack of definitive clinical consensus on evaluation and management of bone fragility in patients with advanced liver disease, the main aim of our study was, therefore, to describe the prevalence, type and site of fragility fractures in a large single-referral center cohort of patients with advanced liver disease undergoing LT, with a complete characterization of etiologies, biochemistry and radiology and secondarily to provide sex-specific information on bone fragility in LT recipients in order to target resources and pharmacologic therapies to this specific setting of patients at exceedingly high risk of bone fracture.

## Patients and methods

### Study population

We performed a single-center retrospective analysis of consecutive patients who underwent LT from January 1st, 2010 to December 31st, 2015 at our referral Liver Transplant Center, IRCSS Azienda Ospedaliero-Universitaria di Bologna, Italy. All patients were evaluated and managed at our center and received a standardized clinical, laboratory and radiological evaluation, including thoracic and abdominal imaging before LT aligned with local and national guidelines. All patients had an abdominal computed tomography (CT) scan performed within 6 months from the LT. Biochemistries, including parathyroid hormone (PTH), minerals, and bone turnover markers (Beta-Cross Laps, CTX and bone-specific alkaline phosphatase, BSAP) were also recorded when available. All recorded biochemistries were taken in a fasting state, between 8.00 and 9.00 a.m. All samples were analyzed at the Unified Metropolitan Laboratory of Bologna [16].

The medical records reviewed were as follows: the Interregional Transplant Association chart (a local chart including clinical data of transplant candidates), as well as the chart of the in-hospital admission at the time of LT surgery, which included the previous relevant clinical history of the patient and comorbidities, including clinical fractures, etiology of liver disease and its complications, biochemistries, radiology reports including bone density tests (dual energy X-ray absorptiometry DXA), as well as concomitant pharmacologic treatments.

The centralized radiological imaging data center (PACS) (the archive that holds all clinically obtained electronic radiological/nuclear medicine images in DICOM® format of each registered patient) was evaluated for each patient. For each patient, a lateral view of a conventional thoracic X-ray and a thoraco-abdominal CT scan (Scout-Scan) performed within 6 months from LT were selected and analyzed. Two trained physicians, bone metabolism specialists (G.Z and P.A), blinded to patient clinical data (except for sex and birth date), including chronic liver disease etiology or severity, re-reviewed all acquired spine images to screen for morphometric vertebral fractures. Inconsistent findings were solved by reaching consensus after several measurements of the vertebra. Semiquantitative visual assessment according to Genant’s criteria [17] was performed to ascertain and assess severity of vertebral deformities. Percentage reductions of either anterior, middle, or posterior vertebral heights were calculated and used to define mild (20–25%), moderate (26–40%) and severe (> 40%) vertebral fractures on lateral projections of spine imaging. Previous kyphoplasty or vertebroplasty were also documented and counted as one or multiple vertebral fractures according to their extension. Date and anatomic site of clinical fragility fractures (i.e. all fractures that would cause a patient to seek medical care, including clinical spine, due to absent or low trauma) were also recorded from the medical records and Interregional Transplant Association charts. DXA scans performed within 2 years of LT were also reviewed, and the lowest value of either femur neck, total hip or lumbar spine BMD T-score was used to classify patients according to Word Health Organization (WHO) BMD categories (normal BMD, osteopenia, or osteoporosis). Both T-scores and Z-scores were reported. Fractured vertebrae (L1–L4) were excluded from the analysis of lumbar BMD T-scores or Z-scores.

### Ethical approval

This study was conducted in line with the Declarations of Helsinki and STROBE (STrengthening the Reporting of OBservational studies in Epidemiology) recommendations [18]. The local Ethical Committee approved this study (protocol code: *16/2020/Oss/AOUBo*).

### Statistical analysis

Absolute numbers and percentages were calculated for categorical data. The results for continuous variables were expressed as means and standard deviation (SD). A comparison of general characteristics of LT recipients was performed by Mann–Whitney *U* test, comparing fractured and non-fractured patients and general characteristics between sexes and within each sex. *χ*^2^ test was used to detect associations between fragility fractures, sex and other clinical data, such as diabetes or corticosteroids use, in both sexes. Multinomial logistic regression with stepwise backward elimination was used to identify risk factors for fragility fractures across the whole population, by adjusting for potential confounders. Covariates were chosen among expected major risk factors for fragility fractures, and significant or near-significant (*P* < 0.10) parameters in simple correlations. Statistical analyses were performed using SPSS (version 26.0). *P* values lower than 0.05 were considered statistically significant.

## Results

Our study identified 429 consecutive patients receiving liver transplantation. After chart review, we excluded 63 patients with transplant surgery due to acute liver failure and no previous history of chronic liver failure, patients not undergoing their first LT (i.e. reoperation) and any combined transplantation (concomitant kidney or heart transplantations). High-trauma fractures (motor vehicle accidents, etc.) were not counted as fragility fractures. All the remaining fractures were considered to be due to low-trauma or osteoporosis, unless otherwise stated. Twenty-six patients (6.0% of the total cohort) had missing or inaccessible radiological imaging/reports and were also excluded. A total of 366 patients were included in the final analysis (Figure S1, supplementary).

### Characteristics of liver transplant recipients: whole population and gender comparison

Of 366 LT recipients included in the study, the majority of them had viral cirrhosis—144 (39.3%)—and 94 (25.7%) had multifactorial disease (Table 1 and 2). Of the 94 patients with multifactorial disease, 61 (64.8%) had viral + alcoholic etiologies, 14 (14.9%) had viral + rare disease etiologies, 13 (13.8%) had viral + MASH etiologies, 1 (1.1%) had cholestatic + viral etiologies, 3 (3.2%) had viral + alcoholic + MASH etiologies, and 2 (2.1%) had alcoholic + MASH etiologies. The overall cohort was composed of 107 (29.3%) women and 259 (70.7%) men, with significant differences in sex prevalence among the etiology categories, with autoimmune and cholestatic disease being more prevalent in women (9.3% and 10.3% vs. 0.1% and 4.2%, respectively), while ALD and multifactorial disease being more common in men (13.9% and 28.6% in men vs. 5.6% and 18.7% in women, respectively). Clinical and anthropometric characteristics are shown in Table 1 and  2. The mean age was 52 years with no significant difference between women and men. A positive smoking history was more frequent in men (26.6% vs. 10.3%, *P* = 0.001). Diabetes and hepatocellular carcinoma were more frequent in men than in women, while females were more frequently exposed to glucocorticoids. Mean body mass index (BMI) was 25.6 kg/m^2^, with no differences between sexes. Hip BMD and estimated glomerular filtration rate (eGFR) were lower in women than in men, while hypertension was similar between the sexes (Table 1 and 2).

**Table 1:** Clinical characteristics of the whole population, according to sex: continuous variables

	Women *N* = 107		Men *N* = 259		Whole population *N* = 366		*P* value
	*N*	Mean ± SD	*N*	Mean ± SD	*N*	Mean ± SD	
Age (years)	107	50.7 ± 11.6	259	53.4 ± 9.9	366	52.6 ± 10.5	0.065
Weight (kg)	103	66.7 ± 13.6	258	78.0 ± 14.4	361	74.7 ± 15.0	** < 0.001**
BMI (kg/m^2^)	96	25.5 ± 4.5	246	25.5 ± 3.7	342	25.5 ± 4.0	0.347
MELD score	106	15.6 ± 9.7	259	16.3 ± 8.5	365	16.1 ± 8.9	0.487
*Laboratory*							
GOT (U/L)	99	94 ± 163	246	79 ± 60	345	83 ± 101	0.297
GPT (U/L)	100	72 ± 211	246	64 ± 116	346	66 ± 150	**0.010**
Total bilirubin (mg/dL)	100	5.6 ± 7.1	248	6.4 ± 14.1	348	6.2 ± 12.5	0.590
GGT (U/L)	99	61.3 ± 57.4	245	86.2 ± 91.4	344	79.0 ± 83.8	**0.004**
ALP (U/L)	98	239 ± 169	246	219 ± 154	344	225 ± 158	0.310
Urea nitrogen (mg/dL)	96	26.6 ± 18.0	243	36.3 ± 33.1	339	33.5 ± 29.9	**0.020**
Glucose (mg/dL)	95	90 ± 33	245	106 ± 40	340	101 ± 39	** < 0.001**
Albumin (g/dL)	98	3.49 ± 0.62	242	3.51 ± 0.67	340	3.50 ± 0.66	0.708
INR	99	1.77 ± 1.06	246	1.56 ± 0.43	345	1.62 ± 0.67	0.326
Platelet count (10^9/L)	100	117.5 ± 120.4	248	92.0 ± 75.4	348	99.3 ± 91.2	0.430
White blood cell (10^9/L)	99	5.07 ± 3.60	246	5.18 ± 3.31	345	5.15 ± 3.39	0.261
Hemoglobin (g/dL)	100	10.89 ± 1.76	246	11.62 ± 2.13	346	11.41 ± 2.05	**0.004**
*Bone metabolism*							
Lumbar BMD (g/cm2)	13	0.83 ± 0.15	27	0.89 ± 0.12	40	0.87 ± 0.13	0.305
Lumbar T-score	13	−2.15 ± 1.25	27	−1.86 ± 1.08	40	−1.95 ± 1.13	0.479
Lumbar Z-score	12	−1.23 ± 1.21	27	−1.43 ± 1.08	39	−1.37 ± 1.11	0.761
Femur Neck BMD (g/cm^2^)	12	0.67 ± 0.12	28	0.75 ± 0.13	40	0.72 ± 0.13	0.087
Femur Neck T-score	12	−1.71 ± 1.05	28	−1.37 ± 0.91	40	−1.47 ± 0.96	0.301
Femur Neck Z-score	12	−0.86 ± 0.89	28	−0.55 ± 0.95	40	−0.65 ± 0.93	0.400
Total Hip BMD (g/cm^2^)	11	0.81 ± 0.17	29	0.93 ± 0.16	40	0.90 ± 0.17	0.058
Total Hip T-score	11	−1.39 ± 1.17	29	−0.72 ± 1.01	40	−0.91 ± 1.08	**0.033**
Total Hip Z-score	11	−0.75 ± 1.14	29	−0.36 ± 1.01	40	−0.47 ± 1.05	0.154
PTH (pg/mL)	8	49 ± 43	19	61 ± 86	27	57 ± 75	0.559
Calcium (mg/dl)	98	8.89 ± 0.66	241	8.82 ± 0.62	339	8.84 ± 0.63	0.513
Phosphate (mg/dL)	82	3.10 ± 0.76	199	3.12 ± 0.71	281	3.11 ± 0.72	0.965
Magnesium (mg/dl)	76	1.95 ± 0.30	189	1.95 ± 0.30	265	1.95 ± 0.30	0.920
Urinary Calcium (mg/24 h)	9	9.02 ± 10.53	23	6.91 ± 6.69	32	7.51 ± 7.83	0.950
Urinary Phosphate (g/24 h)	9	1.08 ± 1.58	19	0.64 ± 0.27	28	0.78 ± 0.91	0.806
25OH Vitamin D (ng/mL)	6	13 ± 8	30	15 ± 7	36	14 ± 7	0.445
Bone specific alkaline phosphatase (BSAP) (microg/L)	2	44.8 ± 27.5	7	29.8 ± 9.9	9	33.1 ± 14.6	0.380
eGFR (mL/min)	99	80.27 ± 25.03	244	86.41 ± 28.57	343	84.64 ± 27.70	**0.010**
Serum creatinine (mg/dL)	98	0.91 ± 0.73	244	1.11 ± 0.84	342	1.06 ± 0.82	** < 0.001**

**Table 2:** Clinical characteristics of the whole population, according to sex: categorical variables

	Women	Men	Overall population	*P* value
	*N* = 107	*N* = 259	*N* = 366	
Fragility fractures	40/107	115/259	155/366	0.216
Ethnicity				
White Caucasian	103	254	357	0.799
Other	1	4	5	
Total	104	258	362	
Arterial hypertension	18/100	55/245	73/345	0.359
Child–Pugh				
Missing	18	27	45	0.064
A	18	55	73	
B	38	70	108	
C	33	107	140	
Total	107	259	366	
Alcohol	17/107_a_	101/259_b_	118/366	** < 0.001**
Smoking	11/107_a_	69/259_b_	80/366	**0.001**
Vitamin D3 supplements	1/107	13/259	14/366	0.064
PPI	9/107_a_	54/259_b_	63/366	**0.004**
Calcium carbonate supplements	1/107	6/259	7/366	0.380
Diabetes	12/107_a_	76/259_b_	88/366	** < 0.001**
Corticosteroid use	14/107_a_	16/259_b_	30/366	**0.028**
Ascites	59/107	142/259	201/366	0.956
Encephalopathy	42/107	97/259	139/366	0.747
Hepatocellular carcinoma	31/107_a_	118/259_b_	149/366	**0.003**
GI Hemorrhage	10/107	28/259	38/366	0.676
Portal vein thrombosis	12/107	29/259	41/366	0.996
Cirrhosis etiology				
Autoimmune hepatitis	10_a_	2_b_	12	** < 0.001**
Cholestatic disease	11_a_	11_b_	22	
Viral	39	105	144	
MASH	0	2	2	
Alcoholic	6_a_	36_b_	42	
Cryptogenic/rare disease	21_a_	29_b_	50	
Multifactorial	20_a_	74_b_	94	
Total	107	259	366	
Vertebral fractures	36/107	109/259	145/366	0.133
Genant’s vertebral fracture grade				
Mild	24	66	90	**0.031**
Moderate	6_a_	37_b_	43	
Severe	6_a_	6_b_	12	
Total	36	109	145	
DXA WHO classification				
Normal BMD	2	5	7	0.838
Low BMD/osteopenia	8	12	20	
Osteoporosis	5	10	15	
Total	15	27	42	

Number. total numbers

Subscript a and b within the same variable express *P* < 0.05

Instead, when we compared patients without fractures (84/211) versus patients with fractures (65/155) no differences occurred about the presence of hepatocellular carcinoma in the two populations (*P* 0.683).

Regarding bone metabolism, fragility fractures prevalence was 155/366 (42.3%) in the overall population, with no significant differences between sexes (fracture prevalence in women was 37.4%, in men was 44.4%). Calcium and vitamin D3 supplements were equally distributed among both sexes, while a small proportion of patients were taking bisphosphonates (13/366, 3.5%). Laboratory parameters of mineral metabolism were tested in very few patients, and no significant differences could be observed between sexes.

Among patients with vertebral fractures (*n* = 145), mild vertebral fractures (Genant grade 1) were the most frequently observed (90/145, 62.1%), with similar prevalence between sexes. Moderate vertebral fractures (Genant grade 2) were more common in men compared to women (33.9% vs 16.7%, *P* < 0.05). Severe (Genant grade 3) vertebral fractures occurred more frequently in women compared to men (16.7% vs. 5.5%, *P* < 0.05). The overall number of patients with clinical fragility fractures (i.e. symptomatic) was 50/366 (13.7%). Of these, most (*n* = 43, 86%) were vertebral fractures. Other clinical fractures were at the humerus (*n* = 1), ribs (*n* = 7), femur (*n* = 1), and clavicle (*n* = 1). The median time between clinical fracture occurrence and LT was 2 months. All the remaining fractures were morphometric vertebral fractures. Fracture prevalence among transplant recipients was stable across each year of the study period (Figure S2, supplementary).

Women with fractures, compared to women without fractures had worse kidney function, lower urinary calcium, lower BMD and more commonly having alcoholic etiology (Supplementary Table a. and b.). Compared to men without fractures, men with fractures had lower 25-OH vitamin D levels, with no other noticeable significant differences in laboratory or clinical data (Supplementary Table c. and d.).

**Fig. 1:**
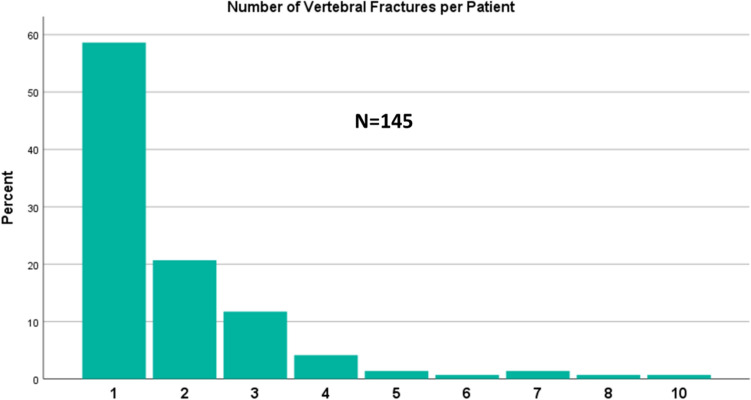
Distribution of single and multiple vertebral fractures across patients with vertebral fractures (N = 145)

Most vertebral fractures were single fractures, although a significant proportion of patients (*n* = 60, 41.3%) had two or more vertebral fractures, up to a maximum of 10 vertebral fractures per patient **(**Fig. 1**)**.

### Characteristics of liver transplant recipients with bone fractures

**Fig. 2:**
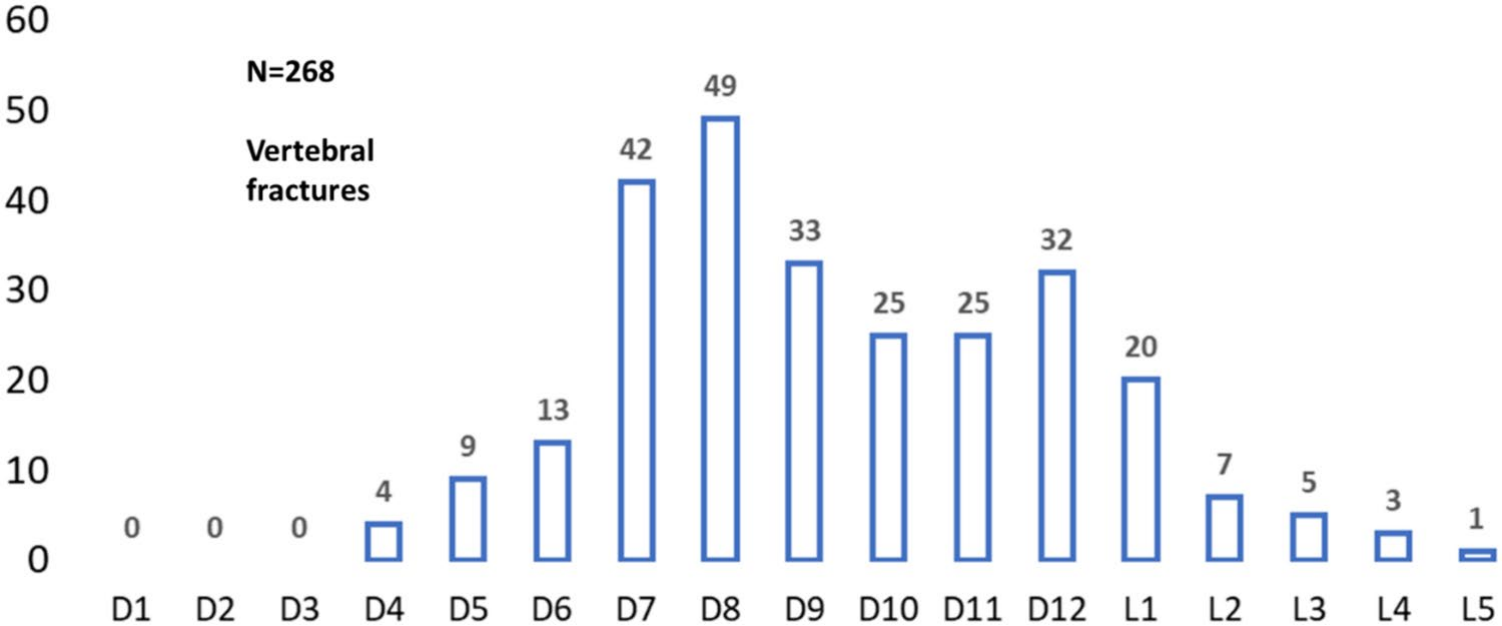
Distribution of vertebral fractures (absolute frequencies)

Patients with bone fractures presented similar age and BMI compared to patients without fractures, although serum glutamic pyruvic transaminase (GPT) was lower and serum creatinine greater than patients with no fractures. Other parameters, both from clinical history and laboratory, were similar between groups (Table 3 and 4). The severity of liver disease was not different between groups. Fragility fractures showed similar rates across each liver disease etiology (Figure S3, supplementary). The most common vertebral sites were at T7, T8, T9 and T12 vertebrae (Fig. 2). Lower rates of fractures were observed in the lumbar spine. The most frequently observed vertebral fracture type was wedge fractures, with a minor but significant proportion of crush or biconcave fractures **(Figure S4, supplementary**).

**Table 3:** Clinical characteristics of the patients with and without fractures: continuous variables

	Patients without fractures *N* = 211		Patients with fractures *N* = 155		Overall *N* = 366	*P* value
	*N*	Mean ± SD	*N*	Mean ± SD		
Age (years)	211	51.8 ± 11.4	155	53.6 ± 8.9	366	0.350
Weight (kg)	207	73.6 ± 14.6	154	76.3 ± 15.5	361	0.104
BMI (kg/m^2^)	194	25.2 ± 3.8	148	26.0 ± 4.1	342	0.053
MELD score	210	15.7 ± 8.7	155	16.6 ± 9.1	365	0.355
*Laboratory*						
GOT (U/L)	197	85 ± 78	148	82 ± 125	345	0.240
GPT (U/L)	196	70 ± 137	150	62 ± 165	346	**0.043**
Total Bilirubin (mg/dL)	198	5.3 ± 7.1	150	7.2 ± 17.1	348	0.624
GGT (U/L)	195	79 ± 71	149	79 ± 97	344	0.263
ALP (U/L)	195	228 ± 154	149	221 ± 164	344	0.271
Urea Nitrogen (mg/dL)	193	34.0 ± 29.6	146	32.9 ± 30.4	339	0.707
Glucose (mg/dL)	193	106 ± 45	147	95 ± 28	340	0.107
Albumin (g/dL)	195	3.48 ± 0.67	145	3.53 ± 0.62	340	0.408
INR	196	1.61 ± 0.58	149	1.62 ± 0.78	345	0.913
Platelet count (10^9^/L)	198	98.78 ± 87.69	150	100.19 ± 95.98	348	0.626
White blood cell (10^9^/L)	196	5.26 ± 3.75	149	4.99 ± 2.84	345	0.948
Hemoglobin (g/dL)	196	11.58 ± 2.04	150	11.17 ± 2.04	346	0.084
*Bone metabolism*						
Lumbar BMD (g/cm^2^)	19	0.885 ± 0.126	21	0.856 ± 0.139	40	0.473
Lumbar T-score	19	−1.82 ± 1.05	21	−2.06 ± 1.20	40	0.424
Lumbar Z-score	19	−1.28 ± 0.89	20	−1.44 ± 1.29	39	0.642
Femur Neck BMD (g/cm^2^)	18	0.748 ± 0.141	22	0.704 ± 0.127	40	0.242
Femur Neck T-score	18	−1.35 ± 1.05	22	−1.57 ± 0.88	40	0.414
Femur Neck Z-score	18	−0.48 ± 0.96	22	−0.77 ± 0.90	40	0.406
Total Hip BMD (g/cm^2^)	19	0.924 ± 0.160	21	0.879 ± 0.182	40	0.273
Total Hip T-score	19	−0.70 ± 1.01	21	−1.08 ± 1.12	40	0.188
Total Hip Z-score	19	−0.28 ± 0.95	21	−0.63 ± 1.12	40	0.180
PTH (pg/mL)	17	72.35 ± 92.16	10	32.60 ± 20.17	27	0.269
Calcium (mg/dL)	190	8.80 ± 0.58	149	8.88 ± 0.68	339	0.325
Phosphate (mg/dL)	158	3.0 ± 0.7	123	3.1 ± 0.6	281	0.836
Magnesium (mg/dL)	143	1.93 ± 0.25	122	1.96 ± 0.33	265	0.585
Urinary calcium (mg/24 h)	11	8.72 ± 9.14	21	6.86 ± 7.21	32	0.427
Urinary phosphate (g/24 h)	9	1.082 ± 1.572	19	0.644 ± 0.294	28	0.768
25OH Vitamin D (ng/mL)	18	16 ± 8	18	12 ± 6	36	0.087
Bone specific alkaline phosphatase (BSAP) (microg/L)	2	22.2 ± 4.3	7	36.3 ± 15.1	9	0.143
Estimated GFR (mL/min)	196	86.9 ± 28.2	147	81.6 ± 26.7	343	0.092
Serum creatinine (mg/dL)	195	1.02 ± 0.84	147	1.09 ± 0.78	342	**0.023**

**Table 4:** Clinical characteristics of the patients with and without fractures: categorical variables

	Without fractures *N* = 211	With fractures *N* = 155	Overall number *N* = 366	*P* value
Ethnicity				
White Caucasian	203	154	357	0.440
Other	5	0	1	
Total	208	154	362	
Arterial hypertension	43/197	30/148	73/345	0.726
Child–Pugh				
Missing	30	15	45	0.508
A	44	29	73	
B	59	49	108	
C	78	62	140	
Total	211	155	366	
Alcohol use	62/211	56/155	118/366	0.173
Smoking	46/211	34/155	80/366	0.975
Vitamin D3 supplements	6/211	8/155	14/366	0.253
PPI	34/211	29/155	63/366	0.516
Calcium carbonate	5/211	2/155	7/366	0.456
Diabetes	56/211	32/155	88/366	0.192
Corticosteroid use	16/211	14/155	30/366	0.617
Ascites	112/211	89/155	201/366	0.410
Encephalopathy	83/211	56/155	139/366	0.532
Hepatocellular carcinoma	84/211	65/155	149/366	0.683
GI Hemorrhage	25/211	13/155	38/366	0.283
Portal thrombosis	27/211	14/155	41/366	0.259
Cirrhosis etiology				
Autoimmune hepatitis	8	4	12	0.698
Cholestatic disease	10	12	22	
Viral	82	62	144	
MASH	1	1	2	
Alcoholic	21	21	42	
Cryptogenic/rare disease	31	19	50	
Multifactorial	58	36	94	
Total	211	155	366	
Vertebral fractures	0	145/155	145/366	** < 0.001**
DXA WHO classification				
Normal BMD	5	2	7	0.369
Low BMD/osteopenia	9	11	20	
Osteoporosis	6	9	15	
Total	20	22	42

### Characteristics of liver transplant recipients: effect of glucocorticoids on metabolic bone disease

Glucocorticoid administration differed across etiologies, although its impact on fragility fractures, vertebral fractures, or bone mineral density by DXA was not evident (Supplementary Table e.).

### Characteristics of liver transplant recipients: effect of diabetes on metabolic bone disease

Diabetes prevalence differed according to etiology, although it was not associated with fragility fractures (*P* = 0.192), vertebral fractures prevalence or severity, or low bone density by DXA (Supplementary Table f.).

### Predictors of bone fragility fractures

A logistic regression model including age, sex, BMI, alcohol use, eGFR, etiology (autoimmune or cholestatic disease vs. other), revealed that only BMI was negatively associated with prevalent fragility fractures (odds ratio, OR 1.058, 95% CI 1.001–1.118, *P* = 0.046), independent of other risk factors. In LT recipients, for each one-unit decrease of BMI, the risk of fragility fractures would increase by 5.8%, and vice versa (Table 5).

**Table 5:** Multinomial logistic regression^a^

				95%CI	
Risk factors for fragility fractures	Beta coefficient	*P* value	Adjusted odds-ratio	Lower bound	Upper bound
Constant	−1.709	0.019			
BMI	−0.056	**0.046**	1.058	1.001	1.118

^a^Backward stepwise logistic regression analysis adjusted for age, sex, alcohol use, estimated GFR, etiology (autoimmune or cholestatic disease vs. other)

## Discussion

Our study investigated the prevalence of fragility fractures, either clinical or morphometric, in a large cohort of patients undergoing LT due to different etiologies, who were fully characterized in terms of radiology and medical history. The type and the most frequent location of such fractures were also assessed in order to generate a consistent fracture risk profile of a modern cohort of LT recipients. The prevalence of osteoporotic fractures within the cohort was 42.3%, most of which were thoracic vertebral fractures, with femur, humerus and ribs fractures uncommon despite the large sample size. Thanks to the blind re-evaluation of the X-rays of the spine and lateral Scout-CTs of the thoraco-lumbar vertebral column, a large proportion of patients with metabolic bone disease at the time of transplantation was noted. Most fractures (88%) were anterior wedge fractures of mild to moderate severity, with women having a more severe phenotype than men, although a slightly higher fracture prevalence was shown in men. For each one-unit decrease in BMI, fragility fracture risk increased by 5.8% independently of age, kidney function, etiology of liver disease and alcohol consumption.

To the best of our knowledge, thanks to the sample size, our is the largest study investigating LT candidates over the last 20 years, thus providing an updated clinical picture of modern cohorts of LT recipients. Studies carried out so far were mostly heterogeneous in terms of fracture prevalence in LT recipients, with a huge range of prevalence which was usually reported somewhere between 3 and 43%. Small studies often suffer from possible selection bias or variability in the criteria used in the definition of metabolic bone diseases such as osteoporosis or low BMD. In the study published in 1997 by Monegal A. et al. [8], conducted on 58 cirrhotic candidates to liver transplant, it was observed that 43% of the patients had osteoporosis that was diagnosed according to at least one vertebral fracture and/or a BMD of the lumbar vertebrae < 2 standard deviations compared to the mean values of healthy subjects of the same age [8]. Wariaghli G. et al., in a 2010 study conducted on 64 patients with chronic liver disease, showed that 45.5% of patients had osteoporosis and only 5.3% had vertebral fractures [9]. This study also came with the limitation of a minimal number of patients. Furthermore, the patients examined had only primary biliary cholangitis (PBC) or viral liver disease, limiting speculations on other etiologies.

Only few studies analyzing bone fracture risk among LT candidates have been published over the last 10 years. A study [19] on 128 patients found that the severity of liver cirrhosis was associated with hip fractures. This study, however, examined a cohort of elderly patients that were more than one decade older than those in our study [19]. Another registry-based study carried out in Sweden assessed fracture risk in MASH, showing a slightly higher rate of fractures [20]. We did not find the same results in our study regarding MASH etiology. A limitation could be represented by the low percentage of patients with MASH (0.2%) in our population, which contradicts the growing trend of this disease in the world population. However, considering the presence of MASH also in patients with multifactorial disease, we achieve almost 20% in the average study population. Given this consideration, we found that the liver disease etiology, however, does not represent a risk factor for an increase in fractures in our transplant cohort. This assumption is supported by the long-term fracture risk shown in the Sweden study population with MASH, which was very similar to the general population [20]. All these studies could therefore suggest that fragility fractures might be predominantly caused by liver dysfunction alone rather than a specific chronic liver disease etiology. As cirrhosis worsens, metabolic bone disease might also worsen, reaching its worst scenario right at the time of transplantation [20]. This hypothesis was confirmed by a recent study which assessed 102 patients before and after LT, finding that malnutrition and low BMI were the main determinants of osteopenia/osteoporosis, regardless of etiology, similarly to our study [21].

Over the years, several studies have investigated metabolic bone disease in subjects with PBC and primary sclerosing cholangitis (PSC). In a 1994 study by Camisaca M. et al., conducted on 25 women with PBC, a rapid BMD loss of 3.5% was observed in only 6 months [22], with no data on fracture prevalence. Eastell et al., in a study conducted on 210 women with PBC, described lower BMD compared to controls, although the prevalence of fractures was not assessed [23]. Angulo et al. in a study of 81 patients with PSC, demonstrated that the lumbar spine BMD of the patients was lower than age- and sex-matched healthy controls, as well as that 3% of patients had fragility fractures. Finally, they observed that patients with fractures had more advanced liver disease [24]. In this study the prevalence of fractures was presumably underestimated as the study also involved patients with PSC in the initial stages of the disease, thereby representing a limitation. Our study, instead, suggests that cholestatic disease might be equally important as a risk factor for fracture as other liver disease etiologies, because bone fracture prevalence was non-significantly different from other non-cholestatic disorders. This data can be explained by the increasing trend in the average age of LT candidates the last decade and, consequently, in their comorbidities [7].

Gallego-Rojo F. J., et al., in a 1998 study conducted on 32 patients with cirrhosis of viral etiology, showed an osteoporosis prevalence by DXA to be up to 50%. The limitations of this study were the small sample of patients and the missing data about fractures [25]. In our study fracture prevalence in viral cirrhosis was noted to be over 40%, consistent with previous findings.

Therefore, by estimating the risk of fractures across various etiologies of liver disease, the present study suggests that liver etiology may not be as critical as it was initially thought, with the long-standing liver disease per se being the main risk factor for prevalent fractures. Fracture prevalence in patients with liver disease and cirrhosis at the time of LT was very high (approximately 42%) and fractures were mainly located in the thoracic vertebrae, independent of age and sex. It is yet uncertain why men had a slightly higher prevalence of fracture, although a predominantly male cohort may have affected this in the absence of other consistent explanations. Selection bias might be another reason behind this slight disproportion. BMI was the only independent predictor of fracture prevalence: for every one-unit decrease in BMI, risk of fractures increased proportionally.

In recent years, the hepatologists’ community has focused increasing interest on sarcopenia and frailty as prevalent complications able to predict morbidity, mortality, poor quality of life and worse post-LT outcomes in patients with cirrhosis. Osteoporosis should be considered an emerging issue associated with sarcopenia in LT candidates. Since these complications are potentially modifiable with early identification and therapy, clinicians should pay attention to accurately recognizing and evaluating both sarcopenia and osteoporosis in LT candidates and carriers [26].

## Strengths and limitations

Strengths of this study are the large sample size, the consecutive enrolment of the patients, the centralized laboratory and full access to radiological imaging, with blinded review of radiological images, as well as the accuracy of a chart review study regarding correct diagnoses. Moreover, the study’s single-center nature attenuated variability in managing of chronic liver disease before LT.

Being a study grounded in clinical practice, limitation of this study is the absence of a control group and the cross-sectional design. Being ours a major referral center, fracture prevalence might be slightly greater than expected, because of a possible higher frequency of more severe chronic liver disease. Unfortunately, most patients had not a complete mineral metabolism evaluation through the laboratory or DXA. A limited sample size might affect the finding of comparable BMD between males and females, although numerically higher BMD was found in males, as expected. This limitation also prevents speculation on underlying bone metabolism and density in these patients, and, therefore, on the best anti-osteoporotic medications to choose in this setting. Moreover, the menopausal status was not available for all women, although a considerable proportion of women were likely premenopausal based on mean age of the population. Last, the retrospective nature of clinical data might also hide some unintentional bias.

## Conclusions

Our study provides substantial evidence, confirming with its large sample size previous findings of a considerable fracture burden in patients awaiting and undergoing LT. These data support the need for a thorough bone metabolism evaluation and management for this category of patients, and implementation of this in future guidelines.

Osteoporotic vertebral fractures are frequent complications of the underlying end-stage disease. In this large study, most patients had one or two vertebral fractures, but a considerable proportion of patients experienced multiple vertebral fractures. The most affected sites are the thoracic vertebrae, in particular T7, T8, T9 and T12, and the most frequent fractures were anterior wedges. Furthermore, the majority of vertebral fractures were asymptomatic, making vertebral morphometry an essential tool to screen for bone fragility. Femoral fractures or other peripheral clinical fractures were uncommon possibly due to a relatively young population, based on mean age at transplantation.

The prevalence of fractures was similar across all etiologies, as opposed to previous limited-sample studies indicating cholestatic liver disorders as one of the etiologies carrying more significant fracture risk. The prevalence of fractures was also similar across different age groups, and comparable between males and females, although severe vertebral fractures were more common in women.

Our findings should be considered in multidisciplinary liver cirrhosis management. Osteoporotic fractures constitute, in fact, the leading risk factor for subsequent bone fractures and significantly influence the quality of life of these patients before and after LT [27]. Liver transplant screening should include laboratory tests relating to bone metabolism, a DXA to quantify bone mass and spine morphometry to exclude the presence of vertebral fractures. Future studies assessing the impact of these parameters on fracture incidence after transplantation will be warranted to estimate fracture risk in the post-transplantation period and its impact on the overall survival of patients receiving liver transplantation.

## Supplementary Information

Below is the link to the electronic supplementary material.

Supplementary file1 (DOCX 236 KB)

## Data Availability

The data that support the findings of this study are available upon reasonable request.

## Abbreviations

ALD: Alcohol-related liver disease
ALP: Total alkaline phosphatase
BMD: Bone mineral density
BMI: Body mass index
BSAP: Bone specific alkaline phosphatase
CI: Confidence interval
CT: Computed tomography
CTX: Beta-CrossLaps
DAAs: Direct antiviral drugs
DXA: Dual energy X-ray absorptiometry
eGFR: Estimated glomerular filtration rate
HBV: Hepatitis B virus
HCV: Hepatitis C virus
GI: Gastrointestinal
GOT: Glutamate oxaloacetate transaminase
GGT: Gamma-glutamyl transferase
GPT: Glutamic pyruvic transaminase
L: Lumbar vertebrae
LT: Liver transplantation
MASLD: Metabolic dysfunction-associated steatohepatitis
MELD: Model for end-stage liver disease
NASH: Non-alcoholic steatohepatitis
OR: Odds ratio
PBC: Primary biliary cholangitis
PPI: Proton-pump inhibitors
PSC: Primary sclerosing cholangitis
PTH: Parathyroid hormone
SD: Standard deviation
WHO: Word Health Organization

## References

[1] Patel N, Muñoz SJ (2015) Bone disease in cirrhosis. Clin Liver Dis 6(4):96–99. 10.1002/cld.498.PMID:31040999;PMCID:PMC6490654 10.1002/cld.498 PMC6490654 31040999

[2] Guañabens N, Parés A (2010) Liver and bone. Arch Biochem Biophys 503(1):84–94. 10.1016/j.abb.2010.05.030. (**Epub 2010 Jun 9 PMID: 20537977**) 20537977 10.1016/j.abb.2010.05.030

[3] Nakchbandi IA (2014) Osteoporosis and fractures in liver disease: relevance, pathogenesis and therapeutic implications. World J Gastroenterol 20(28):9427–9438. 10.3748/wjg.v20.i28.9427.PMID:25071337;PMCID:PMC4110574 25071337 10.3748/wjg.v20.i28.9427 PMC4110574

[4] Compston JE, McClung MR, Leslie WD (2019) Osteoporosis Lancet 393(10169):364–376. 10.1016/S0140-6736(18)32112-3. (**PMID: 30696576**) 30696576 10.1016/S0140-6736(18)32112-3

[5] Nuti R, Brandi ML, Checchia G, Di Munno O, Dominguez L, Falaschi P, Fiore CE, Iolascon G, Maggi S, Michieli R, Migliaccio S, Minisola S, Rossini M, Sessa G, Tarantino U, Toselli A, Isaia GC (2019) Guidelines for the management of osteoporosis and fragility fractures. Intern Emerg Med 14(1):85–102. 10.1007/s11739-018-1874-2. Epub 2018 Jun 13. PMID: 29948835; PMCID: PMC6329834. 10.1007/s11739-018-1874-2 PMC6329834 29948835

[6] Guichelaar MM, Malinchoc M, Sibonga J, Clarke BL, Hay JE (2002) Bone metabolism in advanced cholestatic liver disease: analysis by bone histomorphometry. Hepatology 36(4 Pt 1):895–903. 10.1053/jhep.2002.36357. (**PMID: 12297836**) 12297836 10.1053/jhep.2002.36357

[7] Durand F, Levitsky J, Cauchy F, Gilgenkrantz H, Soubrane O, Francoz C (2019) Age and liver transplantation. J Hepatol 70(4):745–758. 10.1016/j.jhep.2018.12.009. (**Epub 2018 Dec 18 PMID: 30576701**) 30576701 10.1016/j.jhep.2018.12.009

[8] Monegal A, Navasa M, Guañabens N, Peris P, Pons F, Martinez de Osaba MJ, Rimola A, Rodés J, Muñoz-Gómez J (1997) Osteoporosis and bone mineral metabolism disorders in cirrhotic patients referred for orthotopic liver transplantation. Calcif Tissue Int 60(2):148–154. 10.1007/s002239900205. PMID: 9056162. 10.1007/s002239900205 9056162

[9] Wariaghli G, Mounach A, Achemlal L, Benbaghdadi I, Aouragh A, Bezza A, El Maghraoui A (2010) Osteoporosis in chronic liver disease: a case-control study. Rheumatol Int 30(7):893–899. 10.1007/s00296-009-1071-8. (**Epub 2009 Jul 28 PMID: 19636560**) 19636560 10.1007/s00296-009-1071-8

[10] Collier J (2007) Bone disorders in chronic liver disease. Hepatology 46(4):1271–1278. 10.1002/hep.21852. (**PMID: 17886334**) 17886334 10.1002/hep.21852

[11] Compston JE, Vedi S, Kaptoge S, Seeman E (2007) Bone remodeling rate and remodeling balance are not co-regulated in adulthood: implications for the use of activation frequency as an index of remodeling rate. J Bone Miner Res 22(7):1031–1036. 10.1359/jbmr.070407. (**PMID: 17501624**) 17501624 10.1359/jbmr.070407

[12] Eastell R, Rosen CJ, Black DM, Cheung AM, Murad MH, Shoback D (2019) Pharmacological management of osteoporosis in postmenopausal women: an endocrine society clinical practice guideline. J Clin Endocrinol Metab 104(5):1595–1622. 10.1210/jc.2019-00221. (**PMID: 30907953**) 30907953 10.1210/jc.2019-00221

[13] Guañabens N, Parés A (2011) Management of osteoporosis in liver disease. Clin Res Hepatol Gastroenterol 35(6–7):438–445. 10.1016/j.clinre.2011.03.007. (**Epub 2011 May 4 PMID: 21546334**) 21546334 10.1016/j.clinre.2011.03.007

[14] Wilson T, Katz JM, Gray DH (1981) Inhibition of active bone resorption by copper. Calcif Tissue Int 33(1):35–39. 10.1007/BF02409410. (**PMID: 6780154**) 6780154 10.1007/BF02409410

[15] Schiefke I, Fach A, Wiedmann M, Aretin AV, Schenker E, Borte G, Wiese M, Moessner J (2005) Reduced bone mineral density and altered bone turnover markers in patients with non-cirrhotic chronic hepatitis B or C infection. World J Gastroenterol 11(12):1843–1847. 10.3748/wjg.v11.i12.1843.PMID:15793878;PMCID:PMC4305888 15793878 10.3748/wjg.v11.i12.1843 PMC4305888

[16] https://www.ausl.bologna.it/cit/urc/le-carte-dei-servizi-delle-unita-operative/laboratorio-unico-metropolitano-lum/ (last access 08/20/2024).

[17] Genant HK, Wu CY, van Kuijk C, Nevitt MC (1993) Vertebral fracture assessment using a semiquantitative technique. J Bone Miner Res 8(9):1137–1148 8237484 10.1002/jbmr.5650080915

[18] von Elm E, Altman DG, Egger M, Pocock SJ, Gøtzsche PC, Vandenbroucke JP; STROBE Initiative. (2007) The Strengthening the Reporting of Observational Studies in Epidemiology (STROBE) statement: guidelines for reporting observational studies. PLoS Med 4(10):e296. 10.1371/journal.pmed.0040296. PMID: 17941714; PMCID: PMC2020495. 10.1371/journal.pmed.0040296 PMC2020495 17941714

[19] Hundersmarck D, Groot OQ, Schuijt HJ, Hietbrink F, Leenen LPH, Heng M (2022) Hip fractures in patients with liver cirrhosis: worsening liver function is associated with increased mortality. Clin Orthop Relat Res 480(6):1077–1088. 10.1097/CORR.0000000000002088. Epub 2021 Dec 31. PMID: 34978539; PMCID: PMC9263483. 10.1097/CORR.0000000000002088 PMC9263483 34978539

[20] Wester A, Hagström H (2022) Risk of fractures and subsequent mortality in non-alcoholic fatty liver disease: A nationwide population-based cohort study. J Intern Med 292(3): 492–500. 10.1111/joim.13497. Epub 2022 Apr 19. PMID: 35373876; PMCID: PMC9545244. 10.1111/joim.13497 PMC9545244 35373876

[21] Huldén E, Castedal M, Karlsson MK, Kalaitzakis E, Swärd P (2020) Osteoporosis in cirrhotics before and after liver transplantation: relation with malnutrition and inflammatory status. Scand J Gastroenterol 55(3):354–361. 10.1080/00365521.2020.1735507. (**Epub 2020 Mar 17 PMID: 32180479**) 32180479 10.1080/00365521.2020.1735507

[22] Camisasca M, Crosignani A, Battezzati PM, Albisetti W, Grandinetti G, Pietrogrande L, Biffi A, Zuin M, Podda M (1994) Parenteral calcitonin for metabolic bone disease associated with primary biliary cirrhosis. Hepatology 20(3):633–637 (**PMID: 8076921**) 8076921

[23] Eastell R, Dickson ER, Hodgson SF, Wiesner RH, Porayko MK, Wahner HW, Cedel SL, Riggs BL, Krom RA (1991) Rates of vertebral bone loss before and after liver transplantation in women with primary biliary cirrhosis. Hepatology 14(2):296–300 (**PMID: 1860685**) 1860685

[24] Mounach A, Ouzzif Z, Wariaghli G, Achemlal L, Benbaghdadi I, Aouragh A, Bezza A, El Maghraoui A (2008) Primary biliary cirrhosis and osteoporosis: a case-control study. J Bone Miner Metab 26(4):379–384. 10.1007/s00774-007-0833-1. (**Epub 2008 Jul 4 PMID: 18600405**) 18600405 10.1007/s00774-007-0833-1

[25] Gallego-Rojo FJ, Gonzalez-Calvin JL, Muñoz-Torres M, Mundi JL, Fernandez-Perez R, Rodrigo-Moreno D (1998) Bone mineral density, serum insulin-like growth factor I, and bone turnover markers in viral cirrhosis. Hepatology 28(3):695–699. 10.1002/hep.510280315. (**PMID: 9731561**) 9731561 10.1002/hep.510280315

[26] Lai JC, Tandon P, Bernal W, Tapper EB, Ekong U, Dasarathy S, Carey EJ (2021) Malnutrition, Frailty, and Sarcopenia in Patients With Cirrhosis: 2021 Practice Guidance by the American Association for the Study of Liver Diseases. Hepatology 74(3):1611–1644. 10.1002/hep.32049.Erratum.In:Hepatology.2021Dec;74(6):3563.PMID:34233031;PMCID:PMC9134787 34233031 10.1002/hep.32049 PMC9134787

[27] Zavatta G, Clarke BL (2021) Glucocorticoid- and transplantation-induced osteoporosis. Endocrinol Metab Clin North Am 50(2):251–273. 10.1016/j.ecl.2021.03.002. (**PMID: 34023042**) 34023042 10.1016/j.ecl.2021.03.002

