# Supplementary material for: High bone fracture risk in a large modern cohort of liver transplant recipients
Source: Intern Emerg Med. 2024 Sep 27;20(1):139–50. doi: 10.1007/s11739-024-03767-5 (PMC11794375; doi:10.1007/s11739-024-03767-5)
Supplement: Supplementary file 1 — Supplementary file1 (DOCX 236 KB) [file 11739_2024_3767_MOESM1_ESM.docx]

**Supplementary material:**

**Supplementary tables:**

| **Supplementary Table (a). Women with and without fractures: continuous variables.** | | | | | | | |
| --- | --- | --- | --- | --- | --- | --- | --- |
|  | **Without Fractures** | | | **With Fractures** | | | **P Value** |
|  | **N** | **Mean** | **Std. Deviation** | **N** | **Mean** | **Std. Deviation** |  |
| Age | 67 | 49.58 | 12.24 | 40 | 52.60 | 10.34 | 0.298 |
| Body Weight (kg) | 63 | 65.62 | 13.81 | 40 | 68.40 | 13.46 | 0.277 |
| MELD score | 66 | 15.0 | 9.9 | 40 | 16.5 | 9.5 | 0.457 |
| GOT | 60 | 89.08 | 109.12 | 39 | 103.77 | 223.65 | 0.895 |
| GPT | 60 | 62.45 | 104.62 | 40 | 88.33 | 311.26 | 0.683 |
| Total Bilirubin | 60 | 5.08 | 6.84 | 40 | 6.51 | 7.63 | 0.125 |
| GGT | 59 | 63.90 | 49.48 | 40 | 57.50 | 68.06 | 0.188 |
| ALP | 59 | 230.37 | 144.50 | 39 | 253.92 | 202.20 | 0.890 |
| Urea | 58 | 27.26 | 17.67 | 38 | 25.64 | 18.86 | 0.589 |
| Creatinine | 60 | 0.78 | 0.23 | 40 | 1.29 | 2.34 | 0.493 |
| Blood glucose | 57 | 95.91 | 41.15 | 38 | 82.55 | 15.50 | 0.199 |
| Albumin | 59 | 3.52 | 0.63 | 39 | 3.44 | 0.61 | 0.443 |
| INR | 59 | 1.74 | 0.81 | 40 | 1.80 | 1.35 | 0.620 |
| Platelet Count | 60 | 109.12 | 98.26 | 40 | 130.24 | 148.15 | 0.335 |
| White blood cell | 60 | 4.75 | 3.26 | 39 | 5.55 | 4.07 | 0.176 |
| Hemoglobin | 60 | 10.99 | 1.80 | 40 | 10.74 | 1.71 | 0.497 |
| Lumbar BMD | 4 | 0.73 | 0.11 | 9 | 0.88 | 0.15 | 0.089 |
| Lumbar T-score | 4 | -2.98 | 1.03 | 9 | -1.78 | 1.20 | 0.122 |
| Lumbar Z-score | 4 | -1.93 | 0.48 | 8 | -0.88 | 1.33 | 0.172 |
| Femur Neck BMD | 4 | 0.61 | 0.14 | 8 | 0.70 | 0.11 | 0.308 |
| Femur Neck T-score | 4 | -2.18 | 1.28 | 8 | -1.48 | 0.93 | 0.396 |
| Femur Neck Z-score | 4 | -1.20 | 0.85 | 8 | -0.69 | 0.91 | 0.393 |
| Total Hip BMD | 4 | 0.76 | 0.14 | 7 | 0.85 | 0.19 | 0.571 |
| Total Hip T-score | 4 | -1.78 | 0.62 | 7 | -1.17 | 1.39 | 0.506 |
| Total Hip Z-score | 4 | -1.03 | 0.67 | 7 | -0.60 | 1.37 | 0.850 |
| PTH [pg/mL] | 5 | 63.60 | 51.09 | 3 | 26.00 | 5.20 | 0.050 |
| Calcium [mg/dl] | 59 | 8.82 | 0.53 | 39 | 9.01 | 0.81 | 0.295 |
| Phosphate [mg/dL] | 51 | 3.01 | 0.81 | 31 | 3.25 | 0.65 | 0.185 |
| Magnesium [mg/dl] | 46 | 1.99 | 0.25 | 30 | 1.90 | 0.36 | 0.158 |
| Urinary Calcium [mg/24h] | 3 | 19.30 | 11.89 | 6 | 3.88 | 5.09 | **0.039** |
| Urinary phosphate [g/24h] | 3 | 2.06 | 2.75 | 6 | 0.59 | 0.33 | 0.796 |
| 25OH Vitamin D [ng/mL] | 3 | 13.77 | 13.22 | 3 | 12.57 | 3.65 | 0.513 |
| Bone specific alkaline phosphatase (BSAP) [microg/L] | 1 | 25.30 |  | 1 | 64.30 |  | 0.317 |
| Estimated GFR | 61 | 84.59 | 22.32 | 38 | 73.35 | 27.77 | **0.038** |
| Serum Creatinine | 60 | 0.78 | 0.27 | 38 | 1.11 | 1.10 | 0.156 |
| BMI | 57 | 25.23 | 4.50 | 39 | 26.03 | 4.67 | 0.330 |

| **Supplementary Table (b). Women with and without fractures: categorical variables.** | | | | | |
| --- | --- | --- | --- | --- | --- |
| **Variable** | **Category** | **Without Fractures** | **With Fractures** | **Total Number** | **P Value** |
| Ethnicity | White Caucasian | 64 | 39 | 103 | 0.436 |
|  | Other | 1 | 0 | 1 |  |
|  | Total | 65 | 39 | 104 |  |
| Arterial Hypertension |  | 9 | 9 | 18 | 0.339 |
|  | Total | 60 | 40 | 100 |  |
| Child–Pugh | Missing | 14 | 4 | 18 | 0.294 |
|  | A | 13 | 5 | 18 |  |
|  | B | 21 | 17 | 38 |  |
|  | C | 19 | 14 | 33 |  |
|  | Total | 67 | 40 | 107 |  |
| Alcohol use |  | 7_a_ | 10_b_ | 17 | **0.046** |
|  | Total | 67 | 40 | 107 |  |
| Smoking |  | 5 | 6 | 11 | 0.214 |
|  | Total | 67 | 40 | 107 |  |
| Vitamin D3 intake |  | 0 | 1 | 1 | 0.193 |
|  | Total | 67 | 40 | 107 |  |
| PPI |  | 3 | 6 | 9 | 0.058 |
|  | Total | 67 | 40 | 107 |  |
| Calcium carbonate supplements |  | 0 | 1 | 1 | 0.193 |
|  | Total | 67 | 40 | 107 |  |
| Diabetes |  | 9 | 3 | 12 | 0.347 |
|  | Total | 67 | 40 | 107 |  |
| Corticosteroid use |  | 7 | 7 | 14 | 0.295 |
|  | Total | 67 | 40 | 107 |  |
| Ascites |  | 35 | 24 | 59 | 0.435 |
|  | Total | 67 | 40 | 107 |  |
| Encephalopathy |  | 26 | 16 | 42 | 0.903 |
|  | Total | 67 | 40 | 107 |  |
| Hepatocellular carcinoma |  | 21 | 10 | 31 | 0.484 |
|  | Total | 67 | 40 | 107 |  |
| GI Hemorrhage |  | 5 | 5 | 10 | 0.386 |
|  | Total | 67 | 40 | 107 |  |
| Portal thrombosis |  | 8 | 4 | 12 | 0.758 |
|  | Total | 67 | 40 | 107 |  |
| Etiology | Autoimmune | 6 | 4 | 10 | 0.591 |
|  | Cholestatic | 6 | 5 | 11 |  |
|  | Viral | 24 | 15 | 39 |  |
|  | Alcohol | 2 | 4 | 6 |  |
|  | Cryptogenic/rare | 15 | 6 | 21 |  |
|  | Multifactorial | 14 | 6 | 20 |  |
|  | Total | 67 | 40 | 107 |  |
| Vertebral fractures |  | 0_a_ | 36_b_ | 36 | **<0.001** |
|  | Total | 67 | 40 | 107 |  |
| Genant’s grade | Mild | n.a. | 24 | 24 |  |
|  | Moderate | n.a. | 6 | 6 |  |
|  | Severe | n.a. | 6 | 6 |  |
|  | Total | n.a. | 36 | 36 |  |
| DXA WHO classification | Normal BMD | 2 | 0 | 2 | **0.042** |
|  | Low BMD/osteopenia | 1_a_ | 7_b_ | 8 |  |
|  | Osteoporosis | 3 | 2 | 5 |  |
|  | Total | 6 | 9 | 15 |  |

| **Supplementary Table (c). Men with and without fractures: continuous variables.** | | | | | | | |
| --- | --- | --- | --- | --- | --- | --- | --- |
|  | **Without Fractures** | | | **With Fractures** | | | **P Value** |
|  | **N** | **Mean** | **Std. Deviation** | **N** | **Mean** | **Std. Deviation** |  |
| Age | 144 | 52.92 | 11.02 | 115 | 53.99 | 8.43 | 0.767 |
| Body Weight (kg) | 144 | 77.13 | 13.62 | 114 | 79.11 | 15.31 | 0.294 |
| MELD score | 144 | 16.1 | 8.1 | 115 | 16.6 | 9.0 | 0.581 |
| GOT | 137 | 83.45 | 60.80 | 109 | 74.47 | 59.96 | 0.166 |
| GPT | 136 | 73.76 | 150.23 | 110 | 52.82 | 48.49 | **0.027** |
| Total Bilirubin | 138 | 5.53 | 7.37 | 110 | 7.52 | 19.56 | 0.615 |
| GGT | 136 | 85.56 | 78.52 | 109 | 87.08 | 105.84 | 0.578 |
| ALP | 136 | 227.09 | 159.33 | 110 | 210.51 | 148.28 | 0.263 |
| Urea | 135 | 36.95 | 33.20 | 108 | 35.50 | 33.25 | 0.815 |
| Creatinine | 138 | 1.34 | 2.86 | 110 | 1.01 | 0.61 | 0.559 |
| Blood glucose | 136 | 111.28 | 47.11 | 109 | 99.51 | 30.28 | 0.142 |
| Albumin | 136 | 3.47 | 0.70 | 106 | 3.57 | 0.63 | 0.171 |
| INR | 137 | 1.56 | 0.43 | 109 | 1.55 | 0.41 | 0.684 |
| Platelet Count | 138 | 94.30 | 82.67 | 110 | 89.26 | 65.49 | 0.975 |
| White blood cell | 136 | 5.49 | 3.94 | 110 | 4.80 | 2.25 | 0.323 |
| Hemoglobin | 136 | 11.85 | 2.10 | 110 | 11.34 | 2.14 | 0.094 |
| Lumbar BMD | 15 | 0.93 | 0.09 | 12 | 0.84 | 0.13 | 0.054 |
| Lumbar T-score | 15 | -1.51 | 0.85 | 12 | -2.28 | 1.22 | 0.053 |
| Lumbar Z-score | 15 | -1.12 | 0.92 | 12 | -1.82 | 1.18 | 0.071 |
| Femur Neck BMD | 14 | 0.79 | 0.12 | 14 | 0.71 | 0.14 | 0.089 |
| Femur Neck T-score | 14 | -1.11 | 0.89 | 14 | -1.63 | 0.89 | 0.160 |
| Femur Neck Z-score | 14 | -0.29 | 0.91 | 14 | -0.82 | 0.93 | 0.135 |
| Total Hip BMD | 15 | 0.97 | 0.14 | 14 | 0.89 | 0.18 | 0.097 |
| Total Hip T-score | 15 | -0.42 | 0.92 | 14 | -1.04 | 1.03 | 0.066 |
| Total Hip Z-score | 15 | -0.09 | 0.94 | 14 | -0.65 | 1.03 | 0.084 |
| PTH [pg/mL] | 12 | 76.00 | 106.57 | 7 | 35.43 | 23.89 | 0.612 |
| Calcium [mg/dl] | 131 | 8.79 | 0.61 | 110 | 8.85 | 0.64 | 0.567 |
| Phosphate [mg/dL] | 107 | 3.14 | 0.73 | 92 | 3.09 | 0.68 | 0.544 |
| Magnesium [mg/dl] | 97 | 1.91 | 0.26 | 92 | 1.99 | 0.33 | 0.142 |
| Urinary Calcium [mg/24h] | 8 | 4.76 | 3.63 | 15 | 8.06 | 7.73 | 0.478 |
| Urinary phosphate [g/24h] | 6 | 0.59 | 0.25 | 13 | 0.67 | 0.28 | 0.599 |
| 25OH Vitamin D [ng/mL] | 15 | 17.49 | 7.79 | 15 | 12.61 | 6.59 | **0.042** |
| Bone specific alkaline phosphatase (BSAP) [microg/L] | 1 | 19.10 |  | 6 | 31.65 | 9.62 | 0.134 |
| Albumin [g/dl] | 113 | 3.45 | 0.67 | 93 | 3.65 | 0.75 | 0.159 |
| eGFR | 135 | 87.95 | 30.57 | 109 | 84.50 | 25.88 | 0.310 |
| Serum Creatinine | 135 | 1.14 | 0.98 | 109 | 1.09 | 0.65 | 0.202 |
| BMI | 137 | 25.25 | 3.55 | 109 | 25.99 | 4.02 | 0.113 |

| **Supplementary Table (d). Men with and without fractures: categorical variables.** | | | | | |
| --- | --- | --- | --- | --- | --- |
| **Variable** |  | **Without Fractures** | **With Fractures** | **Total** | **P Value** |
| Ethnicity | White Caucasian | 139 | 115 | 254 | 0.514 |
|  | Other | 4 | 0 | 4 |  |
|  | Total | 143 | 115 | 258 |  |
| Arterial Hypertension |  | 34 | 21 | 55 | 0.317 |
|  | Total | 137 | 108 | 245 |  |
| Child–Pugh | Missing | 16 | 11 | 27 | 0.975 |
|  | A | 31 | 24 | 55 |  |
|  | B | 38 | 32 | 70 |  |
|  | C | 59 | 48 | 107 |  |
|  | Total | 144 | 115 | 259 |  |
| Alcohol use |  | 55 | 46 | 101 | 0.767 |
|  |  | 144 | 115 | 259 |  |
| Smoking |  | 41 | 28 | 69 | 0.456 |
|  | Total | 144 | 115 | 259 |  |
| Vitamin D3 intake |  | 6 | 7 | 13 | 0.482 |
|  | Total | 144 | 115 | 259 |  |
| PPI |  | 31 | 23 | 54 | 0.764 |
|  | Total | 144 | 115 | 259 |  |
| Calcium carbonate supplements |  | 5 | 1 | 6 | 0.167 |
|  | Total | 144 | 115 | 259 |  |
| Diabetes |  | 47 | 29 | 76 | 0.192 |
|  | Total | 144 | 115 | 259 |  |
| Corticosteroids |  | 9 | 7 | 16 | 0.957 |
|  | Total | 144 | 115 | 259 |  |
| Ascites |  | 77 | 65 | 142 | 0.624 |
|  | Total | 144 | 115 | 259 |  |
| Encephalopathy |  | 57 | 40 | 97 | 0.428 |
|  | Total | 144 | 115 | 259 |  |
| Hepatocellular carcinoma |  | 63 | 55 | 118 | 0.513 |
|  | Total | 144 | 115 | 259 |  |
| GI Hemorrhage |  | 20 | 8 | 28 | 0.074 |
|  | Total | 144 | 115 | 259 |  |
| Portal thrombosis |  | 19 | 10 | 29 | 0.254 |
|  | Total | 144 | 115 | 259 |  |
| Etiology | Autoimmune Hepatitis | 2 | 0 | 2 | 0.698 |
|  | Cholestatic disease | 4 | 7 | 11 |  |
|  | Viral | 58 | 47 | 105 |  |
|  | MASH | 1 | 1 | 2 |  |
|  | Alcoholic | 19 | 17 | 36 |  |
|  | Cryptogenic/Rare disease | 16 | 13 | 29 |  |
|  | Multifactorial | 44 | 30 | 74 |  |
| Vertebral fractures |  | 0_a_ | 109_b_ | 109 | **<0.001** |
|  | Total | 144 | 115 | 259 |  |
| Genant’s grade | Mild | n.a. | 66 | 66 | n.a. |
|  | Moderate | n.a. | 37 | 37 |  |
|  | Severe | n.a. | 6 | 6 |  |
|  | Total |  | 109 | 109 |  |
| DXA WHO classification | Normal BMD | 3_a_ | 2_a_ | 5 | 0.212 |
|  | Low BMD/osteopenia | 8_a_ | 4_a_ | 12 |  |
|  | Osteoporosis | 3_a_ | 7_a_ | 10 |  |
|  | Total | 14 | 13 | 27 |  |

| **Supplementary Table e. Effect of corticosteroid exposure.** | | | | | |
| --- | --- | --- | --- | --- | --- |
|  |  | **No Corticosteroids**  **N=336** | **Corticosteroid use**  **N=30** | **Total Number**  **N=366** | **P Value** |
|  |  |  |  |  |  |
| Fragility Fractures |  | 141 | 14 | 155 | 0.617 |
|  | Total | 336 | 30 | 366 |  |
| Etiology | Autoimmune Hepatitis | 2_a_ | 10_b_ | 12 | **<0.001** |
|  | Cholestatic disease | 10_a_ | 12_b_ | 22 |  |
|  | Viral | 142_a_ | 2_b_ | 144 |  |
|  | MASH | 2 | 0 | 2 |  |
|  | Alcoholic | 40 | 2 | 42 |  |
|  | Cryptogenic/Rare disease | 48 | 2 | 50 |  |
|  | Multifactorial | 92_a_ | 2_b_ | 94 |  |
|  | Total | 336 | 30 | 366 |  |
| Vertebral Fractures |  | 132 | 13 | 145 | 0.664 |
|  | Total | 336 | 30 | 366 |  |
| DXA WHO classification | Normal BMD | 6 | 1 | 7 | 0.940 |
|  | Low BMD/osteopenia | 16 | 4 | 20 |  |
|  | Osteoporosis | 12 | 3 | 15 |  |
|  | Total | 34 | 8 | 42 |  |
| Subscript a and b within the same variable express P<0.05. | | | | | |

| **Supplementary Table f. Effect of diabetes on fracture risk.** | | | | | |
| --- | --- | --- | --- | --- | --- |
|  |  | **No diabetes**  **N=278** | **Diabetes**  **N=88** | **Total**  **N=366** | **P value** |
|  |  |  |  |  |  |
| **Fragility Fractures** |  | 123 | 32 | 155 | 0.192 |
|  | Total | 278 | 88 | 366 |  |
| **Etiology** | Autoimmune Hepatitis | 10 | 2 | 12 | **0.015** |
|  | Cholestatic disease | 21_a_ | 1_b_ | 22 |  |
|  | Viral | 101_a_ | 43_b_ | 144 |  |
|  | MASH | 2 | 0 | 2 |  |
|  | Alcoholic | 26_a_ | 16_b_ | 42 |  |
|  | Cryptogenic/Rare disease | 42 | 8 | 50 |  |
|  | Multifactorial | 76 | 18 | 94 |  |
|  | Total | 278 | 88 | 366 |  |
| **Vertebral fractures** |  | 117 | 28 | 145 | 0.086 |
|  | Total | 278 | 88 | 366 |  |
| **Genant’s vertebral fracture grade** | Mild | 72 | 18 | 90 | 0.767 |
|  | Moderate | 36 | 7 | 43 |  |
|  | Severe | 9 | 3 | 12 |  |
|  | Total | 117 | 28 | 145 |  |
| **DXA WHO classification** | Normal BMD | 5 | 2 | 7 | 0.666 |
|  | Low BMD/osteopenia | 16 | 4 | 20 |  |
|  | Osteoporosis | 10 | 5 | 15 |  |
|  | Total | 31 | 11 | 42 |  |
| Subscript _a_ and _b_ within the same variable express P<0.05. | | | | | |

**Supplementary figures**

**Figure S1. Flowchart of the patient selection process.**


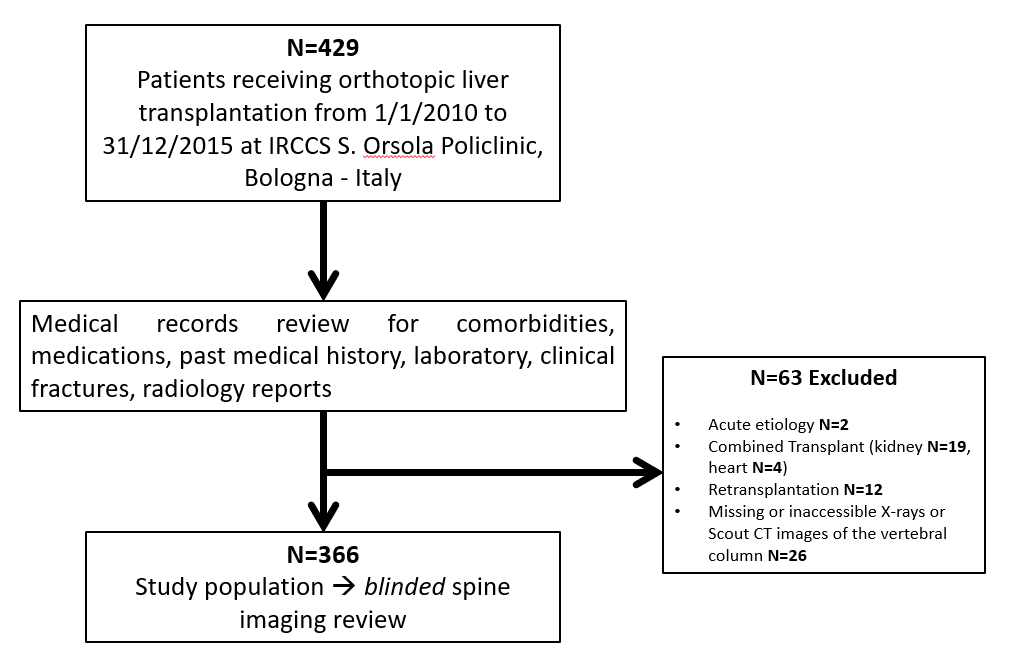


**Figure S2.** Percentage of fractured patients for each calendar year of the study period (P=0.639 for comparison).


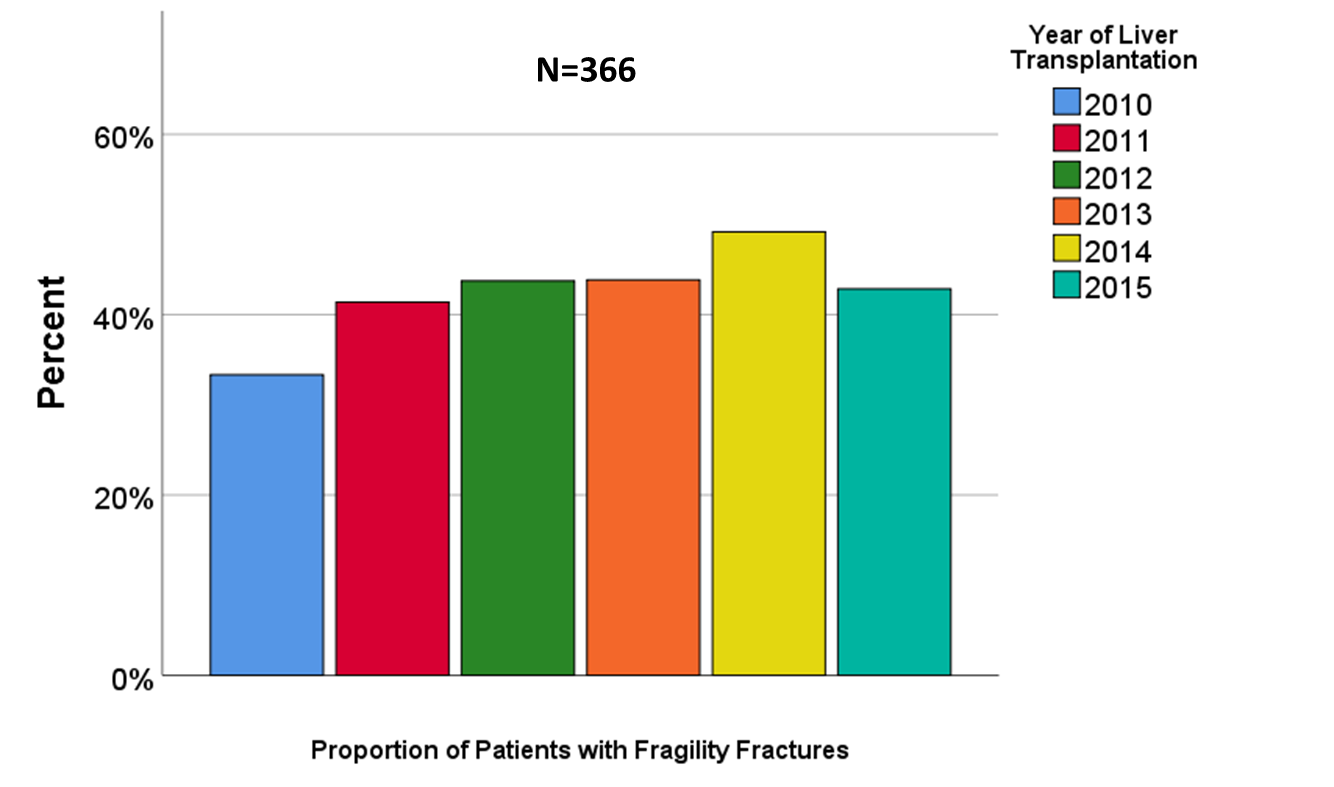


**Figure S3. Percentage of fractured patients in each liver disease etiology (P=0.698).**


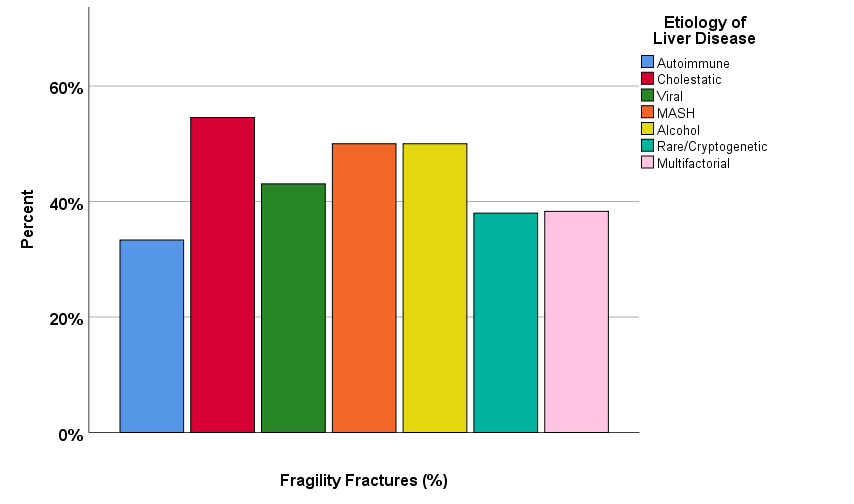


**Figure S4. Types of vertebral fractures.**

**
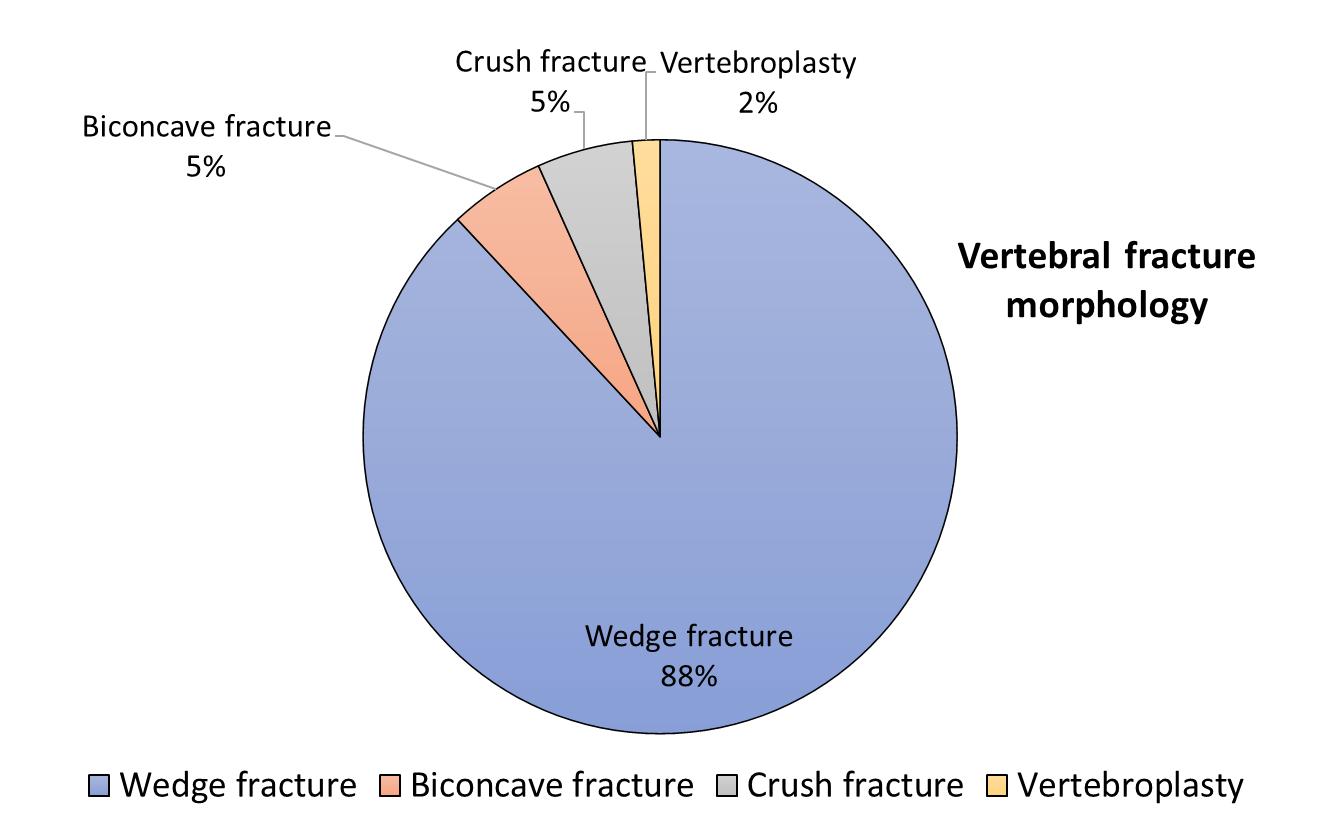
**
